# The Effect of Dicarboxylic Acid Structure on the Plasticizing Ability of Its Ester

**DOI:** 10.3390/polym16233372

**Published:** 2024-11-29

**Authors:** Irina N. Vikhareva, Polina Kruchinina, Dragan Manojlović

**Affiliations:** 1 Nanotechnology REC, South Ural State University, Lenin Prospect 76, 454080 Chelyabinsk, Russia; kruchininap01@gmail.com; 2 Faculty of Chemistry, University of Belgrade, Studentski trg 12-16, 11000 Belgrade, Serbia; manojlo@chem.bg.ac.rs; 3 Department for Ecology and Chemical Technology, South Ural State University, Lenin Prospect 76, 454080 Chelyabinsk, Russia

**Keywords:** adipate, azelate, butoxyethanol, compatibility, efficiency, ester, glutarate, manufacturability, plasticizer, polyvinyl chloride, sebacate

## Abstract

Polyvinyl chloride (PVC) belongs to the most widely used group of thermoplastics. Most of the market for PVC products belongs to plasticized compositions. Plasticizers are the most demanded additives in the polymer industry. Environmental problems and the identified toxicity of the plasticizer di(2-ethylhexyl) phthalate (DEHP) stimulate the restriction of its use and contribute to the development of alternative plasticizers. As a possible replacement for phthalates, esters of dicarboxylic acids are known to provide reduced toxicity and high frost resistance to the resulting compositions. In this regard, esters of dicarboxylic acids and ethoxylated alcohols were obtained and their compatibility with polyvinyl chloride was investigated. The plasticizing effect of the esters obtained was evaluated. The thermomechanical characteristics of PVC compositions containing the developed plasticizers were studied, the glass transition temperature was determined, and the areas of the glassy and highly elastic state of the plastics were identified. It was shown that the chemical structure of dicarboxylate used as a plasticizer determines the important technological and operational characteristics of the PVC plastics obtained. Dibutoxyethyl sebacate (DBES) has the best plasticizing effect among the synthesized esters. The expansion of the highly elastic state area for PVC samples containing this ester exceeded the industrial plasticizers DEHP and di(2-ethylhexyl) adipate (DOA). The indicators of the critical temperature of dissolution of PVC in the esters under study suggest ensuring their low migration from PVC plastics.

## 1. Introduction

Polyvinyl chloride (PVC) is one of the most common polymers due to its affordable cost and high versatility. This material is used in a wide variety of fields, from construction to packaging [1,2,3]. The modification of polyvinyl chloride by introducing necessary additives into the composition is able to meet the growing demand for highly efficient and functional materials. Additives are the main components of most plastics. Plasticizers make up a third of the additive market, of which more than 80% are used in PVC [4,5,6]. Depending on the purpose of the composition in the recipe of the plastic, the content of the plasticizer varies from 10% to 200% by weight of the polymer. The main purpose of plasticizers is to regulate the thermomechanical and rheological properties of the composition to improve the technology. One of the main requirements for PVC additives is the absence of migration from the polymer and the constancy of properties during the entire operation period of the material [7].

In recent decades, plasticizers have been among the most controversial additions to PVC, since there is no absolutely non-migrating plasticizer [8,9,10,11,12,13]. The most widely used plasticizers are phthalates. Their market share is 92%, of which more than 50% of the total production of phthalate surfactants belongs to di(2-ethylhexyl) phthalate (DEHP) [14,15]. The popularity of phthalates is due to their low cost, compatibility with PVC, and excellent plasticization efficiency [16]. The use of the most common dioctyl phthalate is gradually being limited due to potential threats to human health and the environment [17,18,19]. For this reason, measures have been developed to regulate and control the use of phthalate plasticizers [20,21,22]. Safer analogues are becoming increasingly widespread: esters of adipic, sebacic, citric, terephthalic, trimellitic acids, phosphate, cyclohexandicarboxylic esters, and biologically-based alternatives [23,24,25]. However, some of them have limited compatibility with PVC, do not provide a set of properties, or are more expensive.

A wide variety of possible candidates complicates and increases the cost of the experimental development of plasticizers, which is due to the complexity of targeted molecular selection, primarily due to insufficient fundamental knowledge about the mechanism of plasticization. Although plasticizers have been used in the industry for a long time, systematic studies of the effect of the molecular structure of the compound on the effectiveness of plasticizing action have become widespread only recently. For example, in the study referred to in the article, an analysis of the mechanical properties of PVC films plasticized with various succinates and maleates compared with DEHP showed that diester plasticizers achieve optimal efficiency in combination with alcohols containing 4–6 carbon atoms in alkyl chains, while the acidic structure plays a lesser role [26]. In general, the molecular mechanisms underlying plasticization are complex and require careful study.

Several theories have been proposed to explain the mechanism of action of the plasticizer in PVC.

The theory of lubricity suggests that the plasticizer acts as a lubricant, reducing friction and facilitating the movement of polymer chains past each other. Intermolecular forces inside the polymer weaken, reducing the glass transition temperature [27]. In order for the plasticizer to be effective when used in PVC, it must contain two types of structural elements—polar and nonpolar. The polar part of the molecules reversibly attaches to the polymer, thereby softening PVC, and the nonpolar part regulates the interaction, destroying the crystalline regions.

According to gel theory [28], polymer stiffness arises from a three-dimensional grid of weak secondary binding forces along the polymer chains. The plasticizer disrupts the interaction of molecules by embedding between chains and reducing the rigidity of the gel structure. Some plasticizer molecules solvate the polymer at the points of attraction, while other plasticizer molecules cause the rest of the gel structure to swell or expand.

The theory of free volume assumes that there is an internal space inside the polymer [29]. The larger the space, the easier it is for the molecular or polymer chains to move. In the vitreous state, the free volume is small, so the polymer molecules are tightly packed and cannot easily move relative to each other. This makes the polymer rigid and hard. Above the glass transition temperature, thermal energy and molecular vibrations create an additional free volume, which allows polymer molecules to move quickly relative to each other. This effect makes the polymer system more flexible. The free volume can be increased either by modifying the polymer skeleton, for example, by adding a number of side chains or end groups, or by adding small molecules, for example, a plasticizer. Due to the separation of PVC molecules, the glass transition temperature decreases and gives PVC softness and rubber-like properties.

The mechanistic theory of plasticization assumes that the plasticizer molecules are not permanently bound to PVC polymer molecules, but are free and can associate with each other and with polymer molecules in certain areas, for example, amorphous [30]. Since these interactions are weak, a dynamic exchange process is possible when one plasticizer molecule attached to one polymer center can be easily moved or removed using another. Different plasticizers produce different plasticization effects due to the different strength of the plasticizer-polymer and plasticizer-plasticizer interactions. With a small content of plasticizer, the “plasticizer-PVC” interactions prevail, while at high concentrations of plasticizer, the “plasticizer-plasticizer” interactions become more significant. This explains the observed anti-plasticization phenomena, when a low level of plasticizer content increases the rigidity of PVC.

Well-known theories of plasticization, for example, gel theory, free volume theory, and lubricity theory, are rather phenomenological in nature [31,32]. However, the amount of necessary knowledge of the relationship between the molecular structure and the plasticizing ability is insufficient, and the prediction of functional characteristics to guide the work on molecular design is impossible to the full extent.

Molecular modeling in polymers has achieved significant success over the past three decades. One of the fundamental works can be considered the publication by [33]. The analysis showed that the required procedure depends on the type of polymer, and the exact protocol is based on a specific simulated system [34,35]. The creation of models for polymer compositions with additives is a direct continuation of the development of molecular modeling methods. In the near future, computer molecular modeling will undoubtedly be a valuable tool for the development of new plasticizers, guided by predictive plasticizing ability.

Currently, among the well-known industrial alternatives, dicarboxylic acid esters have been widely used, for example, dioctyl adipate and dioctyl sebacate, which, in addition to a reduced adverse effect on living organisms, provide excellent low-temperature characteristics of the materials obtained [36].

Studies of the plasticizing ability of adipate esters in polylactic acid (PLA) have shown that diethyl adipate (DEA) provides the best effect [37]. The cohesive structures of DEA also indicate a resistance to diffusion.

The effectiveness of dibutyl sebacate on an ethyl cellulose polymer has been studied [38]. It was found that dibutyl sebacate is the most successful candidate among the studied sebacates, since the components of the spacers of the DBS aliphatic chain will generate more free volume and increase plasticization.

Succinates have also been studied among biologically-based plasticizers [39] and it was found that molecules with longer alkyl chains being the most effective in reducing Young’s modulus, since they act as a spacer component, providing more movement to polymer chains and free volume in the system [26,40,41]. The presence of esteric groups reduces the tendency to migrate. It is also important to note that at an ester concentration above 30% by weight, a high rate of biodegradation of the polymer composition is ensured [42,43].

The range of applications of dicarboxylic acids is quite extensive. The global market for adipic acid is estimated at about $6.3 billion per year. Therefore, for the purpose of sustainable development, research is underway to develop biological methods of acid synthesis to replace petrochemical raw materials [44,45,46]. Some other members of the geological series are currently successfully obtained on an industrial scale from plant raw materials.

All of the above highlights the need to study the synthesis of new dicarboxylic acid esters, as this class of compounds provides a viable alternative to traditional industrial plasticizers. In addition, the study of their properties is important for practical applications, providing an ideal balance of technological, operational, and health-related properties.

## 2. Materials and Methods

### 2.1. Materials

Industrial samples of suspension polyvinyl chloride supplied by Bashkir Soda Company Caustic JSC (Sterlitamak, Russia) were used as a matrix. Plasticizer dioctyl phthalate (DOP) was supplied by JSC Kamtex-Khimprom (Perm, Russia). The main characteristics are presented in Table 1.

The stabilizer tribasic lead sulfate (TBLS) was manufactured by Baerlocher GmbH (Ingolstadt, Germany). The main characteristics are presented in Table 2.

The stabilizer calcium stearic acid (CaSt) was produced by the HIMSTAB company (Mytishchi, Russia). The main characteristics are presented in Table 3.

Glutaric acid has the form of colorless crystals or a white odorless solid. The mass fraction of the basic substance (%, not less) is 99.0; the melting point is 96 °C. Adipic acid is a white crystalline compound. The mass fraction of the basic substance (%, not less) is 99.8; the melting point is 152.3 °C. Azelaic acid is a white fine crystalline powder. The mass fraction of the basic substance (%, not less) is 99.0; the melting point is 106 °C. Sebacic acid has the form of colorless crystals or a white odorless solid. The mass fraction of the main substance (%, not less) is 99.5; the melting point is 134 °C. Butoxyethanol is a colorless translucent liquid. The main characteristics are presented in Table 4.

### 2.2. Synthesis Methods

The synthesis of esters of dicarboxylic acids and ethoxylated alcohols involves the following. A certain amount of azeotropic toluene solvent, aliphatic acid, the required amount of alcohol, and the calculated amount of paratoluene sulfonic acid catalyst are introduced into a chemical reactor with a connected reverse refrigerator, thermometer, and Dean-Stark trap. The reaction is carried out at the boiling point of the reagent mixture. The control is carried out by the release of the estimated amount of water in the Dean-Stark trap. After the reaction is completed, the flask with the contents is cooled. Then, the esterification is washed sequentially with a 5% alkali solution, water, and a saturated sodium chloride solution and drained with freshly prepared calcined sodium sulfate.

### 2.3. Preparation of Prototypes Containing Plasticizer

All components of the PVC formulation were introduced into a laboratory two-stage mixer model TGHK 5, where they were mixed for 60 min. Two different types of films were obtained from this mixture: rigid and plasticized. The subsequent production of PVC films was carried out using two-roll “SCAMEX” rollers operating at temperatures from 165 to 175 °C for 5–10 min. The specific duration of heat treatment was determined based on the composition of the mixture. After that, pressed samples were obtained by processing on a hydraulic press. Careful control of the temperature and processing time is crucial to achieve the desired characteristics in the final products.

### 2.4. Methods of Analysis

The acid and ether numbers of the plasticizers were determined by the titration of an alcohol solution of ether using a solution of potassium hydroxide in the presence of phenolphthalein.

The refractive index (n) of the esters was measured on a refractometer. The following devices and reagents were used for the test: Abbe type refractometers, a thermostat, a thermometer for measuring temperatures from 0 to 50 °C with a division price of 0.1 °C, ethyl rectified industrial alcohol, or other solvents. The arithmetic mean of two parallel definitions was taken as the test result, the permissible discrepancies between which should not exceed 0.0002.

The density of esters was measured using a general purpose densimeter according to the standard “Liquid resins and plasticizers. Density determination methods”. The essence of the method when using a pycnometer is to determine the mass of resin or plasticizer located in a pycnometer of a known volume at a temperature of (23.0 ± 0.1) °C or at a temperature of (20.0 ± 0.1) °C. This method is suitable for low- and medium-viscosity resins and plasticizers, but there are difficulties when using this method for high-viscosity resins. A pycnometer consisting of a precisely calibrated bulb was used for measurement. The height of the neck of the flask above the mark should not exceed 50 mm. The calibrated volume of the pycnometer in cm^3^ at a temperature of (23.0 ± 0.1) °C or at a temperature of (20.0 ± 0.1) °C, measured by weighing the mass of distilled water in the pycnometer at the appropriate temperature, was calculated to the fourth decimal place. At least two parallel definitions were carried out, the permissible discrepancy between which should not exceed 0.002 g/cm^3^. The density was calculated using the formula.

The mass fraction of volatile compounds was determined according to the standard for plasticizers. A total of (10,000 ± 1000) g of plasticizer was weighed in a cup, previously brought to a constant mass at a temperature of (100 ± 2) °C, and placed on asbestos cardboard in a thermostat, removing the lid. Next, the suspension was kept in a thermal cabinet at a temperature of (100 ± 2) °C for 6 h. The thermometer was installed in the thermal cabinet so that the mercury ball touched the asbestos cardboard. Then, the cup was removed and, closing the lid, placed in a desiccator with calcined chloride calcium and cooled for at least 30 min. The chilled cup was then weighed. The mass fraction of volatile substances as a percentage was calculated using the formula. The arithmetic mean of the results of two definitions was taken as the result of the analysis, the permissible discrepancy between which should not exceed 0.08% absolute.

To analyze the synthesized products, infrared (IR) spectra were obtained through Fourier transform infrared (FTIR) spectroscopy, utilizing potassium bromide (KBr) tablets prepared following the established protocols. The absorption spectra were captured within the wavelength range of 450 to 3700 cm^−1^ using a Shimadzu FTIR-8400S Fourier spectrometer at ambient temperature. The measurements were conducted with a resolution of 4 cm^−1^, and a total of 20 scans were executed for each sample.

The method of dynamic mechanical analysis (DMA) was used to understand the effect of esters on molecular mobility and temperature transitions of plasticized PVC. This was evaluated on a NETZSCH DMA-242 device in stretching mode, in the temperature range from −80 to +100 °C, at a frequency of 1 Hz in an atmosphere of nitrogen supplied at a rate of 100 mL/min. For this purpose, the change in the complex modulus of elasticity was measured at a constant frequency with an increase in the temperature of the polymer sample. The sensitivity of the method to molecular movements makes it possible to determine the temperature transitions of a polymer sample at a fixed frequency over a wide temperature range.

## 3. Results and Discussion

**Scheme 1:**
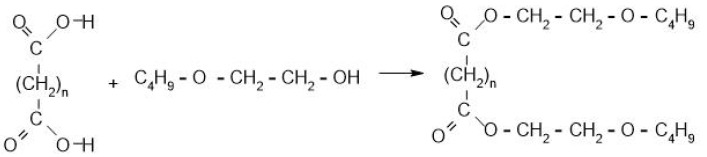
Scheme for the production of dibutoxicarboxylates, where n = 3, 4, 7, 8.

Through the esterification of dicarboxylic acids with ethoxylated butanol according to the previously described method, the following were obtained: dibutoxyethyl glutarate (DBEG), dibutoxyethyl adipate (DBEA), dibutoxyethyl azelate (DBEAz), dibutoxyethyl sebacate (DBES) [3,4] (Scheme 1).

Toluene was used as an azeotropic solvent. Paratoluenesulfonic acid in an amount of 5% of the reaction mass was used as a catalyst. The synthesis was controlled using the amount of water released in the Dean-Stark nozzle. The yield of diesters exceeded 84%. The duration of synthesis was more influenced by the chemical structure of the acid, which was maximally manifested when using adipic acid. With an increase in the length of the chain, that is, with the transition of azelaic acid to sebacic acid, the effect of the steric factor weakened. As a result, the maximum synthesis time of the four studied acids was observed when dibutoxyethyl adipate was obtained. The choice of dicarboxylic acids was based on the expediency of using the resulting ester and the availability of raw materials.

**Figure 1:**
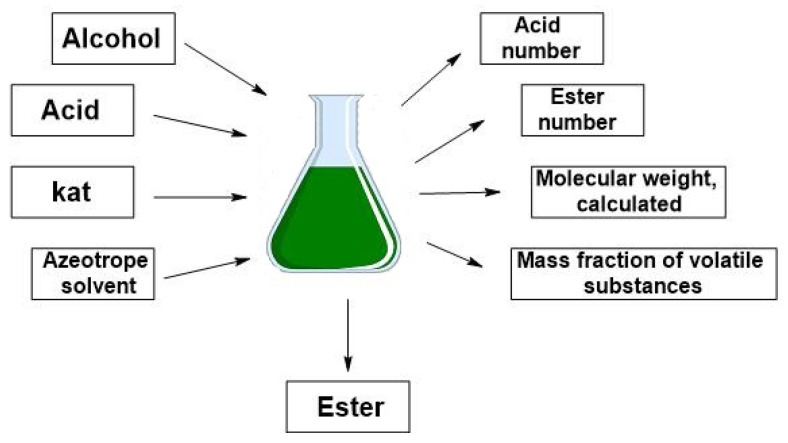
The methodology of choosing a promising ester.

The methodology of choosing a promising ester is presented in Figure 1.

The characteristics of the esters obtained are shown in Table 5.

The dicarboxylates produced exhibited low acid numbers and minimal mass fractions of volatile substances. These properties are crucial for the production of materials based on polyvinyl chloride (PVC), as they directly influence the performance and longevity of the final products. Maintaining low acid numbers is essential to ensure that the PVC materials retain their desired characteristics over time, which is vital for applications requiring durability and reliability. In the context of PVC-based materials, these indicators not only enhance the quality of the products, but also contribute to their overall service life. A lower mass fraction of volatile substances reduces the likelihood of degradation during storage and use, further extending the lifespan of the finished items. This focus on optimizing these chemical properties is aligned with current trends in material science, where sustainability and performance are increasingly prioritized in product development. The resulting dibutoxy acid derivatives are oily yellowish liquids.

**Figure 2:**
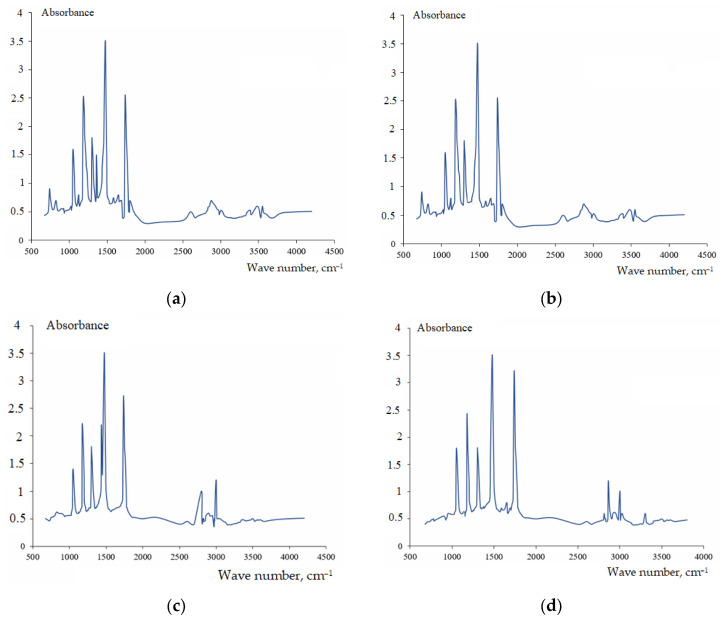
IR spectra of the esters. (**a**) The IR spectrum of dibutoxyethyl glutarate. (**b**) The IR spectrum of dibutoxyethyl adipate. (**c**) The IR spectrum of dibutoxyethyl azelate. (**d**) The IR spectrum of dibutoxyethyl sebacate.

The structure of the obtained dibutoxyethyl derivatives was confirmed by the IR spectra (Figure 2).

The spectra did not exhibit the typical absorption bands associated with the valence vibrations of the carbonyl group found in aliphatic dicarboxylic acids, specifically in the ranges of 1685–1687 cm^−1^ and 1740–1650 cm^−1^. However, an absorption band at 1737 cm^−1^ was present, which is indicative of the carbonyl group in esters. Additionally, the presence of esters was further supported by an absorption band at 1173 cm^−1^ in the IR spectra, corresponding to the vibrations of the C-O-C ether group. Two prominent bands observed at 1330 and 1050 cm^−1^ were associated with the asymmetric vibrations of the carbonyl bond.

**Figure 3:**
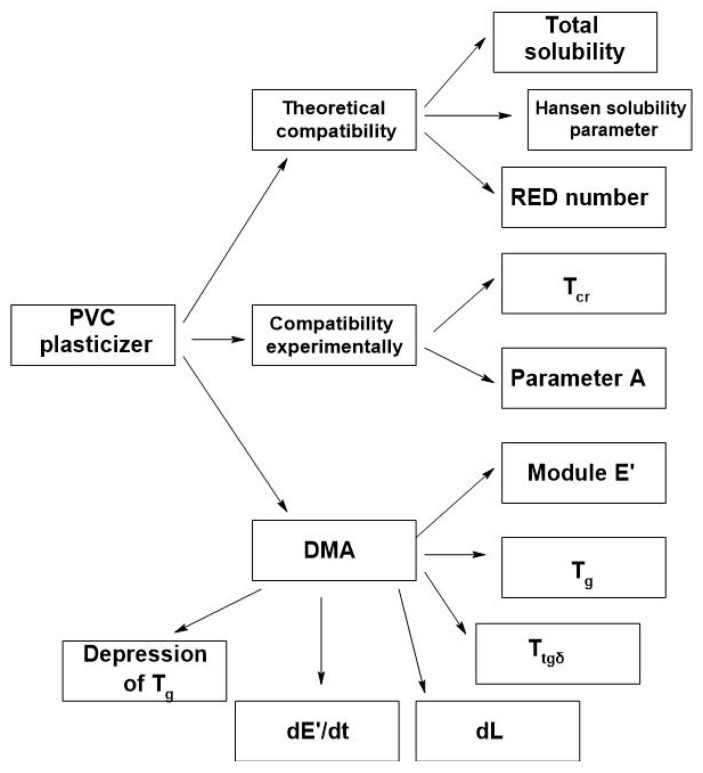
The general scheme of methods for the study of esters.

Synthesized esters were investigated for potential use as PVC plasticizers. The general scheme of the methods used is shown in Figure 3.

A well-functioning plasticizer should provide a balance between compatibility, efficiency, lack of migration, and consequently, the durability of the products used [47]. For this reason, the acceptability of a compound for use as a plasticizer is due to the following characteristics: odorless, environmentally friendly, low volatility, compatibility, plasticizing effect at low temperatures, absence of sweating, and extraction into contacting media [48].

In practical applications, the compatibility of the plasticizer with the polymer is paramount. This compatibility is largely determined by the molecular structure of the plasticizer, particularly the presence and proportion of polar groups within its chemical makeup. Such structural attributes influence how well the plasticizer can integrate with the polymer matrix, affecting both the performance and longevity [49].

There are several theories considering the mechanisms of lubrication: the theory of lubricity, the theory of gel, and the theory of free volume [50,51]. The theory of free volume [52] says that at a temperature above the glass transition, as the temperature decreases, the free volume continuously decreases, but below the glass transition temperature, the diffusion inside the polymer slows down, causing an excess volume. The addition of a plasticizer increases the movements of polymer chains and the available free volume, thereby reducing the glass transition temperature. The branched molecular structure but low molecular weight increases the movements of the main components of the polymer chain with increasing temperature.

**Figure 4:**
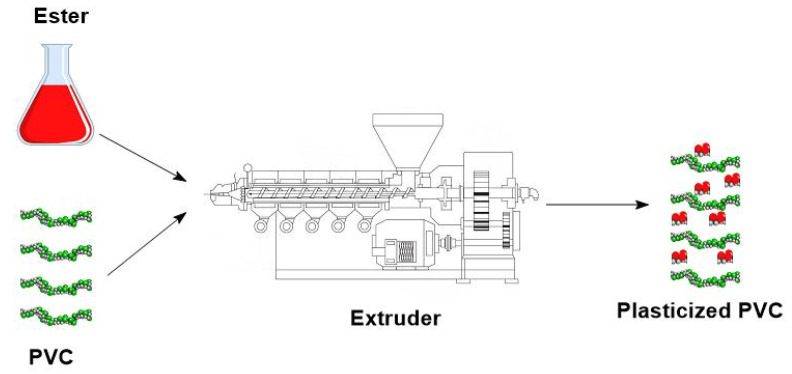
Plasticization of PVC.

The properties of plasticized PVC strongly depend on the interaction of the plasticizer molecule and the polymer. Polar groups lead to the mutual attraction of polymer chains, increasing the glass transition temperature, while large nonpolar side chains keep chains at a distance, reducing the glass transition temperature (Figure 4) [27,28,29,30].

At the molecular level, polyvinyl chloride (PVC) segments interact through robust polar forces arising from the presence of chlorine. This strong interaction is essential for maintaining the structural integrity of PVC but can also limit its flexibility. To enhance the material’s properties, esters are introduced as plasticizers [28]. These esters possess a dual structure characterized by both polar ether groups and nonpolar aliphatic sites, which play a significant role in modifying the polymer’s behavior. The incorporation of plasticizers effectively diminishes the polar interactions between PVC segments, thereby facilitating their movement. This reduction in intermolecular attraction allows for greater flexibility and adaptability of the polymer chain, which is crucial for various applications. Additionally, these plasticizers engage with the polar groups within the PVC matrix, ensuring their retention and contributing to the overall physico-mechanical properties of the material. This interaction not only enhances the flexibility, but also helps maintain the desirable mechanical characteristics, making PVC more versatile for industrial applications. The balance between polar and nonpolar interactions is critical for optimizing the performance of PVC composites, particularly in environments where mechanical strength and durability are essential.

Through migration or evaporation, the plasticizer is gradually separated from the polymer material. The main factors influencing the migration of plasticizers are [30]:

- The type of polymer, its molecular weight, and compatibility with the plasticizer;
- The type and concentration of the plasticizer, its molecular weight, structure, and polarity.

Due to the large number of factors, the theoretical prediction of the migration rate of the plasticizer into the surrounding environment is very difficult, and experiments are almost inevitable. The migration rate doubles for every 7 °C with an increase in temperature [53]. The mechanism governing the migration of plasticizers in polyvinyl chloride (PVC) is significantly influenced by the compatibility between the plasticizer and the polymer matrix. A lower degree of interaction between the plasticizer and PVC, along with reduced compatibility limits, tends to enhance the migration rate of the plasticizer. Specifically, when the concentration of plasticizer within the polymer is maintained below 10%, it becomes fully solvated by the PVC, complicating the extraction of plasticizer molecules and subsequently slowing down the migration process. Conversely, increasing the concentration of plasticizer within the mixture leads to a rise in fragile bonds within the polymer–plasticizer system, which facilitates greater migration rates.

For PVC compositions containing plasticizer levels ranging from 15% to 35%, a nearly linear correlation could be observed between the amount of plasticizer and its migration quantity. This relationship underscores the critical role that the compatibility parameters play in managing both the structural characteristics of the plasticizer and the functional properties of the final material. By optimizing these compatibility parameters, it becomes possible to effectively regulate issues related to high volatility and the surface migration of plasticizers, thereby preserving the operational characteristics.

In practical applications, understanding these dynamics is essential for enhancing material performance and ensuring safety in products that utilize PVC as a primary component.

Plasticizers containing two types of structural elements—polar and nonpolar—are the most effective. The polar part of the molecule is able to reversibly attach to the polymer, thereby softening PVC, while the nonpolar part of the plasticizer molecule allows PVC interactions to be regulated in such a way that they do not reach the force that is capable of destroying crystalline regions. Such groups also add free volume, contribute to screening effects, and provide plasticity. In the case of the esters obtained, the polar components are carbonyl groups C=O; nonpolar—aliphatic chains of ether. The balance between the polar and non-polar parts of the molecules is very important for regulating the solvent effect: if the plasticizer is too polar, it can destroy the crystalline regions of PVC; if it is too nonpolar, incompatibility occurs. When the polymer and plasticizers are heated, the plasticizer begins to solvate PVC molecules. Initially, these interactions only occur in the amorphous part of the polymer, however, with prolonged heating and stirring, PVC crystallites melt, which begin to interact with plasticizers. Upon cooling, a small amount of crystals begins to squeeze out the plasticizer, and the amorphous part of the polymer remains bound to the plasticizer, which increases its volume, lubricates PVC chains, and promotes the formation of a swollen flexible polymer structure [31,54].

A method for determining the solubility parameter is a tool for evaluating the compatibility of a plasticizer.

The calculation of Hansen solubility parameters (HSP—Hansen solubility parameters) [55,56], based on thermodynamic compatibility, serves as a preliminary theoretical assessment of the plasticizing effect of the substance in relation to PVC.

Previously, thermodynamic compatibility was mainly taken into account only during the conversion. However, the toxicity of phthalate plasticizers and their migration have shown the accuracy of calculating the parameters. Plasticizers with a high affinity for the original polymer are less prone to migration from a thermodynamic point of view. The speed of the migration process is also important. However, thermodynamic compatibility gives an idea of how the migration of this plasticizer occurs in comparison with others.

The calculated values of the solubility parameters, the total solubility of Ra, and the number of RED for all of the obtained diesters are shown in Table 6.

Empirically, the proximity of solubility parameters is often used to assess the miscibility of components. A RED number less than 1.0 indicates a high affinity of the plasticizer and polymer, and with a further increase, the compatibility decreases. The calculated values of the individual solubility parameters for all dibutoxyethyl derivatives and dicarboxylic acids were slightly lower than the limit δ_d_ = 15.4 for PVC. For DBEA, this parameter was significantly different and fell out of sequence. For all of the obtained compounds, the RED number was less than 1, which implies their good compatibility with PVC [56]. In general, the dependence of the calculated parameters on the length of the alkyl chain was observed, since longer alkyl chains lead to an increase in the proportion of nonpolar parts in the molecule, which reduces the binding energy.

A practical method for determining the compatibility of a compound with polyvinyl chloride is to establish the critical temperature of polymer decomposition in a plasticizer [57].

In the manufacturing of plasticized PVC products, optimal processability is achieved when temperatures exceed the critical dissolution temperature of the plasticizer by 20–80 °C. When the critical polymer solution temperature falls between 90–135 °C, the plasticizer demonstrates excellent compatibility, allowing for processing at temperatures reaching up to 160 °C. Conversely, if the critical dissolution temperature is within the range of 136–160 °C, the plasticizer exhibits reduced solvent capacity for the polymer and is typically utilized in conjunction with the primary plasticizers. To evaluate the compatibility of these compounds against the standard plasticizer dioctyl phthalate, a parameter known as A was calculated using Formula (1):

A = T_DOP_/T_cr_ × 100%, (1)

where T_DOP_ is the critical dissolution temperature of polyvinyl chloride in the plasticizer DOP; T_cr_ is the critical temperature of dissolution of polyvinyl chloride in the investigated plasticizer.

The results are presented in Table 7.

The obtained values of the critical dissolution temperature and parameter A were in good agreement with the Hansen solubility parameters calculated above. Among the studied compounds, dibutoxyethyl glutarate had the least destructive ability. This compatibility indicator for dibutoxyethyl sebacate differed slightly and amounted to 147 °C. The maximum value of the critical dissolution temperature was characterized by dibutoxyethyl adipate, which indicates its better compatibility with this polymer. Thus, the obtained dibutoxyethyl derivatives of dicarboxylic acids can theoretically be used as secondary plasticizers for elastic materials and PVC-based products.

**Figure 5:**
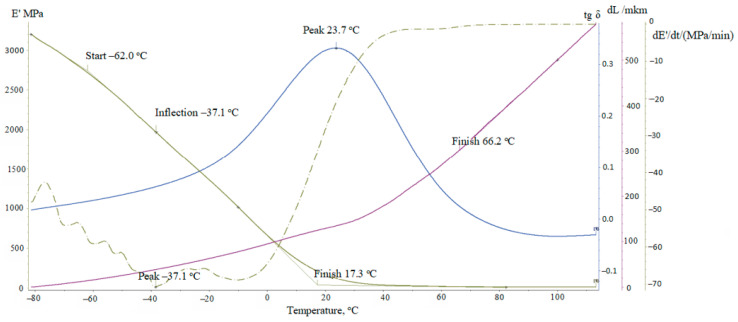
Thermogram of the DMA for PVC, plasticized by DBEG.

**Figure 6:**
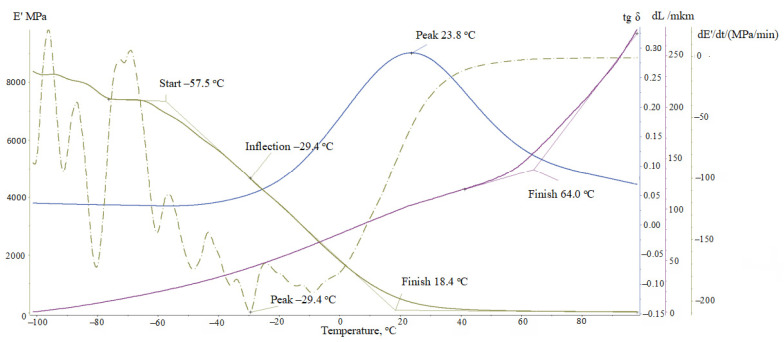
Thermogram of the DMA for PVC, plasticized by DBEA.

**Figure 7:**
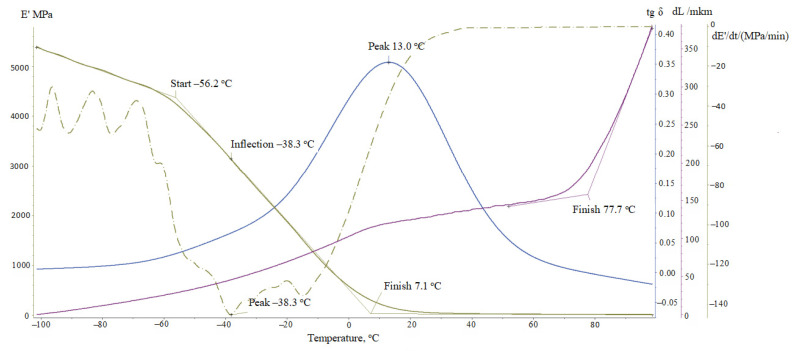
Thermogram of the DMA for PVC, plasticized by DBEAz.

**Figure 8:**
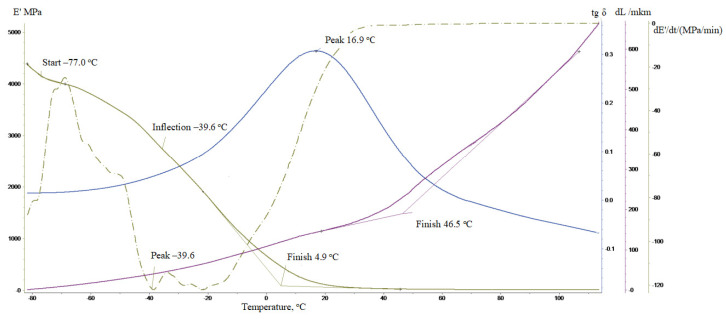
Thermogram of the DMA for PVC, plasticized by DBES.

The main purpose of plasticizers for PVC is to increase the flexibility and manufacturability, which is achieved by reducing the glass transition temperature [58]. The glass transition temperature of plasticized PVC shows the preservation of the elasticity of the polymer material at low temperatures, and the temperature range of the polymer transition from a glass-shaped state to a highly elastic one is key in assessing the manufacturability of a composition containing the studied compound as a plasticizer [59]. To assess the effect of synthesized compounds on the properties of PVC materials, compositions were composed in the following ratio: PVC—100, plasticizer—40, TBLS—2, calcium stearate—1.5, diphenylolpropane—0.25. Plasticized compositions using industrial plasticizers dioctyl phthalate and dioctyl adipate were used as control samples. Dynamomechanical analysis was used to evaluate the effect of the synthesized compounds on the molecular mobility and temperature changes of the plasticized compositions (Figure 5, Figure 6, Figure 7 and Figure 8, Table 8).

Single transitions of the tangent of the mechanical loss angle, denoted as tgδ, were evident in the thermograms of the DMA compositions that incorporated synthesized esters. This observation suggests a uniformity within the plasticized system and indicates the lack of a secondary incompatible phase. The characteristics of the plasticizer play a crucial role in influencing the thermal behavior of PVC materials, as illustrated in Table 4. The transition from a glassy state to a highly elastic state represents a fundamental relaxation process, prominently featured in the main relaxation region known as α-relaxation. At this juncture lies the glass transition temperature, which marks a significant decline in the mobility of polymer molecular segments. Furthermore, the tangent of the mechanical loss angle, tga, which is calculated as the ratio of the loss modulus to the elastic modulus under tensile stress, provides insight into the energy dissipated by the system when subjected to external loads. This parameter effectively quantifies how the material responds to deformation and stress [60].

A study of the thermomechanical characteristics of PVC compositions showed that for samples containing DBEG, DBEAz, and DBES, the transition to a highly elastic state was observed at glass transition temperatures noticeably lower than for compositions containing DBEA. The minimum glass transition temperature of all of the studied samples was 39.6 °C, plasticized by DBES. The results obtained are in good agreement with the Hansen solubility parameters and the parameters of compatibility of diesters with PVC and are of an extreme nature for DBEA. In general, these indicators exceeded the value of the glass transition temperature for plastic with the industrial plasticizer DOP, but noticeably higher than for the samples with the industrial plasticizer DOA.

Heating of the polymer material leads to its softening and a decrease in the dynamic modulus of elasticity E’. The observed changes in properties during heating corresponded to the thermal transitions of the polymer from a glassy state to a highly elastic one. The structure of the studied dibutoxy esters had a direct effect on the modulus of elasticity of the PVC compositions at the beginning of the dynamomechanical tests: for DBEG, the value was comparable to that of PVC plastic with DOA while DBEAz occupied an intermediate position between the values of samples with DOP and DOA. The temperature intervals of transition from a glassy state to a highly elastic one for the PVC compositions with DBES and industrial DOA were almost equal: 81.9 and 81.6 °C, respectively. For a composition with DBEA, this interval was 75.9 °C, which was significantly more than with the industrial plasticizer DOP at 52.6 °C. The maximum transition interval from a glassy state to a highly elastic one was observed for PVC compositions with DBEG at 79.3 °C. In general, plasticizers act by destroying secondary bonds between polymer chains and forming weaker bonds between the polymer and the plasticizer, which increases the mobility of the polymer chain. Intermolecular interactions are functions of the interatomic distances between molecules; the length of the alkyl chain and the structure of the classifier molecule significantly affect its effectiveness [61].

## 4. Conclusions

Esterification of dibasic carboxylic acids with ethoxylated alcohols produced esters with a yield of more than 84%. The physico-chemical characteristics of the obtained compounds confirmed the possibility of testing them as potential PVC plasticizers in order to ensure the required level of performance and the operation period of the finished products. The assessment of key technological and operational parameters for esters and their compositions revealed that all tested diesters exhibited essential compatibility characteristics with polyvinyl chloride (PVC). Additionally, these diesters maintained a critical dissolution temperature suitable for PVC applications, which ensures their low migration from PVC plastics as well as good manufacturability during processing. In general, the solubility and compatibility parameters with respect to polyvinyl chloride of the studied compounds depend on the acid component of the structure of dibutoxy esters and increase with the lengthening of its chain, with the exception of dibutoxyethyl glutarate. The results with a high degree of correlation were confirmed by experimental values of the critical dissolution temperature and calculated parameters of Hansen solubility.

A study of the thermomechanical characteristics of PVC compositions showed that the expansion of the highly elastic state region for PVC samples containing dibutoxyethyl carboxylates was much higher than the reference plasticizer DOP and practically corresponded to the sample containing the industrial analogue DOA. This indicator was even slightly higher for a plasticized sample with a DBES content. However, the stack temperatures of the studied compositions were characterized by reduced values in comparison with the compositions with DOA, but were significantly higher than for compositions with DOP. The data obtained indicate a steady tendency to increase the plasticizing properties of dicarboxylic acid esters as the aliphatic chain of the acid component in the ether lengthens in the range of compounds studied. The aliphatic structure of ether significantly improved the effectiveness of its plasticizing action and technology in the processing process, expanding the temperature range of the highly elastic state, increasing the frost resistance of compositions and reducing the viscosity of polymer melts.

## Figures and Tables

**Table 1:** The main characteristics of dioctyl phthalate (DOP).

Indicator Name	DOP
Acid number, mg KOH/g	0.07
Ester number, mg KOH/g	289
Mass fraction of volatile substances, %, no more	0.1
Flash point, °C	205

**Table 2:** The main characteristics of the stabilizer tribasic lead sulfate (TBLS).

Indicator Name	TBLS
Lead content, %	89 ± 2
pH	7.0–7.5
Ash content, %	at least 95
Appearance	powder

**Table 3:** The main characteristics of the stabilizer calcium stearic acid (CaSt).

Indicator Name	CaSt
Acid number, mg KOH/g	2
Calcium content, %, in the range of	6.4–7.5
Calcium oxide content, %, in the range of	9.0–10.5

**Table 4:** The main characteristics of butoxyethanol.

Indicator Name	Butoxyethanol
Mass fraction of the basic substance, %, not less	99.5
Density at 20 °C, g/cm^3^, within	0.900–0.904
Mass fraction of water, %, not more	0.1
Mass fraction of the acid, in terms of acetic acid, %, no more	0.005
Boiling point, °C, within	169–172

**Table 5:** The main characteristics of the dicarboxylates.

Indicator Name	DBEG	DBEA	DBEAz	DBES
Acid number, mg KOH/g	0.23	0.15	0.19	0.19
Ester number, mg KOH/g	307	321	289	279
Molecular weight, calculated	332	346	388	402
Mass fraction of volatile substances, %, no more	0.3	0.25	0.21	0.18
Yield, %	87.0	87.5	86.0	84.0
Reaction time, min	120	150	145	140
Maximum reaction temperature, °C	118	130	120	120

**Table 6:** Parameters of solubility, total solubility, and RED number for the dibutoxyethyl derivatives.

Compound	δ_d_, (MJ/m^3^)^1/2^	δ_p_, (MJ/m^3^)^1/2^	δ_h_, (MJ/m^3^)^1/2^	δ, (MJ/m^3^)^1/2^	R_A_	RED
DBEG	15.20	8.30	4.90	17.96	7.65	0.93
DBEA	15.29	8.06	4.80	17.94	7.07	0.85
DBEAz	15.17	8.26	4.92	17.89	7.16	0.87
DBES	15.17	8.26	4.92	17.87	7.33	0.89

**Table 7:** Parameters of the compatibility of diesters with PVC.

Compound	T_cr_, °C	Parameter A, %
DBEG	148	81
DBEA	138	87
DBEAz	141	85
DBES	147	83

**Table 8:** Thermo-analytical characteristics of the plasticized PVC compositions obtained by the DMA method.

Plasticizer	E, MPa	T_s_, °C	T_g_, °C	T_f_, °C	T_tgδ_ max, °C	T_f_–T_s_
DOP	5800 (−80 °C)	−29.0	−26.2	23.2	30.0	52.6
DOA	3350 (−80 °C)	−78.4	−65.7	3.2	24.0	81.6
DBEG	3200 (−80 °C)	−62.0	−37.1	17.3	23.7	79.3
DBEA	7650 (−80 °C)	−57.5	−29.4	18.4	23.8	75.9
DBEAz	5000 (−80 °C)	−56.2	−38.3	7.1	13.0	63.3
DBES	4400 (−80 °C)	−77.0	−39.6	4.9	16.9	81.9

T_s_ is the temperature of the beginning of the transition from a glassy state to a highly elastic one; T_g_ is the glass transition temperature; T_f_ is the temperature of the end of the transition from a glassy state to a highly elastic one; T_f_–T_s_ is the temperature range corresponding to the α–relaxation region; E is the modulus of elasticity.

## Data Availability

Dataset available on request from the authors due to privacy.

## References

[1] PVC Applications//PVC—A Circular Material for the Future. https://pvc.org.

[2] Plastics Europe (2022). Plastics the Facts, An Analysis of European Plastics Production, Demand and Waste Data.

[3] Mazitova A.K., Aminova G.K., Vikhareva I.N. (2021). Designing of green plasticizers and assessment of the effectiveness of their use. Polymers.

[4] Vikhareva I.N., Aminova G.K., Mazitova A.K. (2021). Ecotoxicity of the adipate plasticizers: Influence of the structure of the alcohol substituent. Molecules.

[5] Mijangos C., Calafel I., Santamaría A. (2023). Poly (vinyl chloride), a historical polymer still evolving. Polymer.

[6] Vikhareva I.N., Aminova G.K., Mazitova A.K. (2021). Study of the rheological properties of PVC composites plasticized with butoxyethyl adipates. ChemEngineering.

[7] Vikhareva I.N., Aminova G.K., Mazitova A.K. (2022). Development of a highly efficient environmentally friendly plasticizer. Polymers.

[8] Wang X., Song M., Liu S., Wu S., Thu A.M. (2020). Analysis of phthalate plasticizer migration from PVDC packaging materials to food simulants using molecular dynamics simulations and artificial neural network. Food Chem..

[9] Fasake V., Shelake P.S., Srivastava A., Dashora K. (2021). Characteristics of different plastic materials, properties and their role in food packaging. Curr. Nutr. Food Sci..

[10] Cao X.L., Sparling M., Zhao W., Arbuckle T.E. (2021). GC-MS analysis of phthalates and Di-(2-thylhexyl) Adipate in Canadian human milk for exposure assessment of infant population. J. AOAC Int..

[11] Kim J.H., Kim S.H., Lee C.H., Nah J., Hahn A. (2003). DEHP Migration Behavior from Excessively Plasticized PVC Sheets. Bull. Korean Chem. Soc..

[12] Dedov A.V., Chernousova N.V. (2017). Efficiency of reducing the rate of desorption of plasticizer from polyvinyl chloride. Plast. Masses.

[13] Fiala F., Steiner I., Kubesch K. (2000). Migration of di-(2-ethylhexyl)phthalate (DEHP) and diisononyl phthalate (DINP) from PVC articles. Deut. Leb.-Rundsch..

[14] Murphy J. (2001). Additives for Plastics Handbook.

[15] Wadey B.L., Meyers R.A. (2003). Plasticizers. Encyclopedia of Physical Science and Technology.

[16] Godwin A.D. (2017). Plasticizers. Applied Plastics Engineering Handbook.

[17] Mondal T., Mondal S., Ghosh S.K., Pal P., Soren T., Pandey S., Maiti T.K. (2022). Phthalates—A family of plasticizers, their health risks, phytotoxic effects, and microbial bioaugmentation approaches. Environ. Res..

[18] Gholaminejad A., Mehdizadeh G., Dolatimehr A., Arfaeinia H., Farjadfard S., Dobaradaran S., Bonyadi Z., Ramavandi B. (2024). Phthalate esters pollution in the leachate, soil, and water around a landfill near the sea, Iran. Environ. Res..

[19] Luo Q., Liu Z.H., Yin H., Dang Z., Wu P.X., Zhu N.W., Lin Z., Liu Y. (2019). Global review of phthalates in edible oil: An emerging and nonnegligible expo sure source to human. Sci. Total Environ..

[20] Kim S., Kim Y., Moon H.B. (2021). Selective enrichment of antibiotic resistance genes and pathogens on polystyrene microplastics in landfill leachate. Sci. Total Environ..

[21] Ventrice P., Ventrice D., Russo E., De Sarro G. (2013). Phthalates: European regulation, chemistry, pharmacokinetic and related toxicity. Environ. Toxicol. Pharmacol..

[22] US EPA (2014). Appendix A to 40 CFR, Part 423–126 Priority Pollutants.

[23] Bywall L., Cederlund S. (2020). Analysis of Market and Requirements of Plasticizers for Flexible PVC-with a Focus on Perstorp’s Non-Phthalate Plasticizer Pevalen. Master’s Thesis.

[24] Liu Z. (2022). Current Status of Plasticizer Research. Green Catalytic Hydrogenation of Phthalate Plasticizers.

[25] Ito Y., Nakamura T., Yanagiba Y., Ramdhan D.H., Yamagishi N., Naito H., Kamijima M., Gonzalez F.J., Nakajima T. (2012). Plasticizers may activate human hepatic peroxisome proliferator-activated receptor α less than that of a mouse but may activate constitutive androstane receptor in liver. PPAR Res..

[26] Erythropel H.C., Shipley S., Börmann A., Nicell J.A., Maric M., Leask R.L. (2016). Designing green plasticizers: Influence of molecule geometry and alkyl chain length on the plasticizing effectiveness of diester plasticizers in PVC blends. Polymer.

[27] Ambrogi V., Carfagna C., Cerruti P., Marturano V. (2017). Additives in polymers. Modification of Polymer Properties.

[28] Aiken W., Alfrey T., Janssen A., Mark H. (1947). Creep behavior of plasticized vinylite VYNW. J. Polym. Sci..

[29] Lodge T.P., Hiemenz P.C. (2020). Polymer Chemistry.

[30] Ait-Touchente Z., Khellaf M., Raffin G., Lebaz N., Elaissari A. (2024). Recent advances in polyvinyl chloride (PVC) recycling. Polym. Adv. Technol..

[31] Daniels P.H. (2009). A brief overview of theories of PVC plasticization and methods used to evaluate PVC-plasticizer interaction. J. Vinyl. Addit. Technol..

[32] Wypych G. (2017). Plasticizers use and selection for specific polymers. Handbook of Plasticizers.

[33] Theodorou D.N., Suter U.W. (1985). Detailed molecular-structure of a vinyl polymer glass. Macromolecules.

[34] Mavrantzas V.G., Boone T.D., Zervopoulou E., Theodorou D.N. (1999). End-bridging Monte Carlo: A fast algorithm for atomistic simulation of condensed phases of long polymer chains. Macromolecules.

[35] Karayiannis N.C., Mavrantzas V.G., Theodorou D.N. (2002). A novel Monte Carlo scheme for the rapid equilibration of atomistic model polymer systems of precisely defined molecular architecture. Phys. Rev. Lett..

[36] Mazitova A.K., Aminova G.K., Vikhareva I.N. (2022). Development of new environmentally friendly plasticizers. Socar Proc..

[37] Shirai M.A., Grossmann M.V.E., Mali S., Yamashita F., Garcia P.S., Müller C.M.O. (2013). Development of Biodegradable Flexible Films of Starch and Poly(lactic acid) Plasticized with Adipate or Citrate Esters. Carbohydr. Polym..

[38] Mahnaj T., Ahmed S., Plakogiannis F. (2010). Evaluating the Efficacy of a Group of Nontraditional Plasticizers on the Glass Transition Temperature of Ethyl Cellulose Polymer. Drug Dev. Ind. Pharm..

[39] Erythropel H.C. (2015). Evaluation of Maleate, Fumarate, and Succinate Diesters as Potential Green Plasticizers. Doctoral Dissertation.

[40] Erythropel H.C., Brown T., Maric M., Nicell J.A., Cooper D.G., Leask R.L. (2015). Designing Greener Plasticizers: Effects of Alkyl Chain Length and Branching on the Biodegradation of Maleate Based Plasticizers. Chemosphere.

[41] Erythropel H.C., Dodd P., Leask R.L., Maric M., Cooper D.G. (2013). Designing green plasticizers: Influence of Alkyl Chain Length on Biodegradation and Plasticization Properties of Succinate Based Plasticizers. Chemosphere.

[42] Stuart A., LeCaptain D.J., Lee C.Y., Mohanty D.K. (2013). Poly(vinyl chloride) Plasticized with Mixtures of Succinate Diesters—Synthesis and Characterization. Eur. Polym. J..

[43] Elsiwi B.M., Garcia-Valdez O., Erythropel H.C., Leask R.L., Nicell J.A., Maric M. (2020). Fully Renewable, Effective, and Highly Biodegradable Plasticizer: Di-*n*-heptyl Succinate. ACS Sustain. Chem. Eng..

[44] Niu W., Draths K.M., Frost J.W. (2002). Benzene-Free Synthesis of Adipic Acid. Biotechnol. Prog..

[45] Bart J.C.J., Cavallaro S. (2015). Transiting from adipic acid to bioadipic acid. Part II. Biosynthetic pathways. Ind. Eng. Chem. Res..

[46] Corona A., Biddy M., Vardon D., Birkved M., Hauschild M.Z., Beckham G.T. (2018). Life cycle assessment of adipic acid production from lignin. Green Chem..

[47] Kerber M.L., Vinogradov V.L., Golovkin G.S., Gorbatkina Y.A. (2008). Polymer Composite Materials: Structure, Properties, Technology.

[48] Mathews G. (2024). PVC: Production, Properties and Uses.

[49] Winkler R.G., Gompper G. (2020). The physics of active polymers and filaments. J. Chem. Phys..

[50] Harmandaris V.A., Adhikari N.P., van der Vegt N.F.A., Kremer K. (2006). Hierarchical modeling of polystyrene: From atomistic to coarse-grained simulations. Macromolecules.

[51] Peter C., Kremer K. (2009). Multiscale simulation of soft matter systems−from the atomistic to the coarse-grained level and back. Soft Matter.

[52] White R.P., Lipson J.E.G. (2016). Polymer Free Volume and Its Connection to the Glass Transition. Macromolecules.

[53] Grossman F. (2009). Guidelines for the Development of PVC-Based Compositions.

[54] Barstein R.S., Barstein V.I., Kirillovich Y., Nosovsky E. (1982). Plasticizers for Polymers.

[55] Hansen C.M., Beerbower A., Standen A. (1971). Solubility Parameters. Kirk-Othmer Encyclopedia of Chemical Technology, Supplement Volume.

[56] Hansen C.M. (2007). Hansen Solubility Parameter. A User’s Handbook.

[57] Semchikov Y.D. (2003). High-Molecular Compounds.

[58] Bocqué M., Voirin C., Lapinte V., Caillol S., Robin J. (2016). Petro-based and bio-based plasticizers: Chemical structures to plasticizing properties. J. Polym. Sci. Part. A Polym. Chem..

[59] Daniels P.H., Cabrera A. (2015). Plasticizer compatibility testing: Dynamic mechanical analysis and glass transition temperatures. J. Vinyl. Addit. Technol..

[60] Howick C.J. (2000). Plasticizers. Kirk-Othmer Encyclopedia of Chemical Technology.

[61] Pandey S., Kumar V., Kumar P. (2021). Application and analysis of machine learning algorithms for design of concrete mix with plasticizer and without plasticizer. J. Soft Comput. Civil. Eng..

